# Adrenal Tumor Mimicking Non-Classic Congenital Adrenal Hyperplasia

**DOI:** 10.3389/fendo.2020.526287

**Published:** 2020-09-29

**Authors:** Wen-Hsuan Tsai, Chian-Huey Wong, Shuen-Han Dai, Chung-Hsin Tsai, Yi-Hong Zeng

**Affiliations:** ^1^ Division of Endocrinology and Metabolism, Department of Internal Medicine, Mackay Memorial Hospital, Taipei, Taiwan; ^2^ Department of Obstetrics and Gynecology, Mackay Memorial Hospital, Taipei, Taiwan; ^3^ Department of Pathology, Mackay Memorial Hospital, Taipei, Taiwan; ^4^ Department of General Surgery, Mackay Memorial Hospital, Taipei, Taiwan; ^5^ Department of Medicine, MacKay Medical College, New Taipei City, Taiwan

**Keywords:** infertility, 17-OHP, non-classic congenital adrenal hyperplasia, androgen, adrenal tumor

## Abstract

Elevated 17-hydroxyprogesterone may be caused by congenital adrenal hyperplasia, ovarian or adrenal tumors. A positive cosyntropin stimulation test result for 17-hydroxyprogesterone may be found in functional or non-functional tumors and be related to tumor size. Here, we present a case of a 36-year-old woman with a 4-year history of infertility. Laboratory test results revealed elevated progesterone and 17-hydroxyprogesterone, with normal luteinizing hormone, follicle-stimulating hormone, estrogen, testosterone, and anti-Mullerian hormone levels. The 250-μg cosyntropin stimulation test revealed a 17-hydroxyprogesterone level of 11.3 ng/ml (34.3 nmol/L) and 31.8 ng/ml (96.2 nmol/L) at 0 and 60 min, respectively. Non-classic congenital adrenal hyperplasia was diagnosed initially; however, genetic testing revealed no 21-hydroxylase deficiency. She received dexamethasone but progesterone and 17-hydroxyprogesterone levels remained high. Abdominal computed tomography found a 4.5 × 4.8-cm left adrenal tumor. Subsequent pathological report was compatible with an adrenal cortical adenoma. Progesterone and 17-hydroxyprogesterone levels returned to the normal range postoperatively and the 250-μg cosyntropin stimulation test of 17-hydroxyprogesterone showed a normal response. When biochemically diagnosed NCCAH demonstrate no typical features and show poor response to steroid, the patient should undergo gene mutation analysis and receive adrenal or ovarian imaging. For women suffering from infertility, adrenalectomy of 17-OHP secreting adrenal tumor may improve fertility outcome.

## Introduction

Androgen excess is one of the etiologies of infertility and may present as polycystic ovary syndrome (PCOS), ovarian tumor, non-classic congenital adrenal hyperplasia (NCCAH), Cushing’s syndrome, or adrenal tumor. Elevation of plasma 17-OHP concentration above 100 nmol/L can be due to CAH, an adrenal tumor or ovarian source (1). Many decades of elevated ACTH secretion may lead to hyperplasia of the adrenal cortex and tumor formation subsequently. The frequency of biochemically diagnosed CAH in adrenal incidentaloma (AI) cohorts was 5.9%, while only 0.8% was genetically diagnosed as CAH. The discrepancy in biochemical and genetic diagnosis indicated low specificity of 17-OHP levels (2). After exclusion of patients with a 17-OHP level indicating classic CAH, both basal and stimulated 17-OHP by ACTH correlated with the adrenal tumor size. Moreover, this exaggerated response disappeared in most patients after adrenalectomy (2). One-third of biochemically diagnosed CAH patients with AIs had bilateral tumors. As for the group with genetically confirmed CAH, more than half had bilateral AIs (2). CAH patients with AIs may not only have increased basal 17-OHP but also increased basal 11-deoxycortisol, progesterone, and 11-deoxycorticosterone, depending on the steroidogenic pathway involved (3).

Many individuals with 17-OHP levels in the range of 30 to 45 nmol/L have CYP21A2 mutation carrier status, which account for about 10% of patients with AIs (2, 4). In other words, an elevated level of 17-OHP still calls for gene mutation analysis. CYP21A2 mutation carriers may have adrenal tumors with no typical CAH features. However, for those 17-OHP secreting adrenal tumors without CYP21A2 abnormalities, the mechanism of hormone over-secretion and adrenal gland tumor formation is not clear.

Hence, we reported a case initially diagnosed as NCCAH with an adrenal tumor that showed significant remission of high 17-OHP and progesterone after adrenalectomy. We had obtained informed consent from the patient for publication of the submitted article and accompanying images.

## Case Report

This patient was a 36-year-old woman who had a 4-year history of infertility. Menarche took place when she was 13 years old. She complained of irregular menstrual cycle after she went to work at around 25 years old. She visited a gynecologist in 2015 due to prolonged vaginal bleeding and failure to conceive. Physical examination showed no moon face, no buffalo hump, no central obesity, no hirsutism, nor acanthosis nigricans. Initial laboratory tests for evaluation of infertility in 2015 were listed in **Table 1**. She had a history of hyperprolactinemia (27.32 ng/ml, 1–27 ng/ml) and Magnetic Resonance Imaging (MRI) of brain revealed no pituitary lesion. Mild elevation of TSH level was noted, and Anti-TSH receptor antibody, Anti-Thyroid Peroxidase antibody, and anti-thyroglobulin antibody were negative. Thyroxine was prescribed for subclinical hypothyroidism. Gynecologic sonography revealed no adnexal masses with an endometrial thickness of 0.64 cm. Anti-Mullerian hormone level adjusted for age (0.52 ng/ml, 0.07–7.35 ng/ml) indicated poor ovarian reserve. Since she had no history of endometrioma, teratoma, autoimmune disorders, oncological or radiation therapies, tobacco smoking, or increased alcohol consumption, whether low AMH was age or stress related was not clear. Due to above reason, she started *in vitro* fertilization (IVF) after comprehensive counseling. Menstrual cycle was initially regulated using conjugated estrogen 0.625 mg and medroxyprogesterone 5 mg per day. She received several cycles of IVF as anticipated. However, significantly poor ovarian response with persistent elevated progesterone level on the day of trigger was noted despite different ovarian stimulation protocols, which was uncommon in patients closely monitored by IVF specialist. Serial follow-up gynecologic sonography revealed no adnexal masses with persistent thin endometrium (endometrial thickness of 0.38 cm). Further investigation in 2018 showed high level of progesterone and 17-hydroxyprogesterone (**Table 1**). The cosyntropin stimulation test revealed a 17-OHP of 11.3 ng/ml (34.3 nmol/L) and 31.8 ng/ml (96.2 nmol/L) at 0 and 60 min, respectively. Non-classic congenital adrenal hyperplasia (NCCAH) was diagnosed, although genetic testing revealed no CYP21A2 mutation. Dexamethasone was prescribed initially and shifted to prednisolone at another clinic for a total duration of 8 months. Nonetheless, a high progesterone level of 11 ng/ml was still noted.

**Table 1:** Baseline laboratory test.

2015 Infertility workup		
White blood cell count	6,500	(4,000–10,000)/µl
Hemoglobin	12.8	(11–16) g/dl
Platelet count	201,000	(140,000–450,000)/µl
Creatinine	0.9	(0.4–1.2) mg/dl
Alanine aminotransferase	13	(14–40) IU/L
Sodium	142	(136–144) mEq/L
Potassium	4.0	(3.5–5.1) mEq/L
Prolactin	27.32	(1–27) ng/ml
Luteinizing hormone	4.88	follicular phase 3.0–18.6 mIU/ml,
Estrogen	10.81	follicular phase 21–251 pg/ml
Progesterone	2.10	<0.10–0.30 ng/ml
TSH	4.27	0.25–4 U/ml
Free T4	1.39	0.89–1.79 ng/dl
Testosterone	0.51	<0.95 ng/ml
Dehydroepiandrosterone sulphate	102	195.00–507.00 µg/dl
Anti-Mullerian hormone	0.52	0.07–7.35 ng/ml
**2018 Workup for elevation of 17-OHP and adrenal tumor**		
Cortisol	5.03	9.52–26.21 µg/dl
Adrenocorticotropic hormone	8.42	10–70 pg/ml
Plasma renin activity	2.24	0.6–4.18 ng/ml/h
Aldosterone	18.7	4.83–27 ng/dl
Urine 17-hydroxycorticosteroids	8.2	2–8 mg/day
Urine 17-ketosteroids	13.8	6–14 mg/day
Urine vanillylmandelic acid	2.8	1–7.5 mg/day
Urine dopamine	332.5	50–450 µg/day
Urine epinephrine	26.8	<22.4 µg/day
Urine norepinephrine	61	12.1–85.5 µg/day

**Figure 1:**
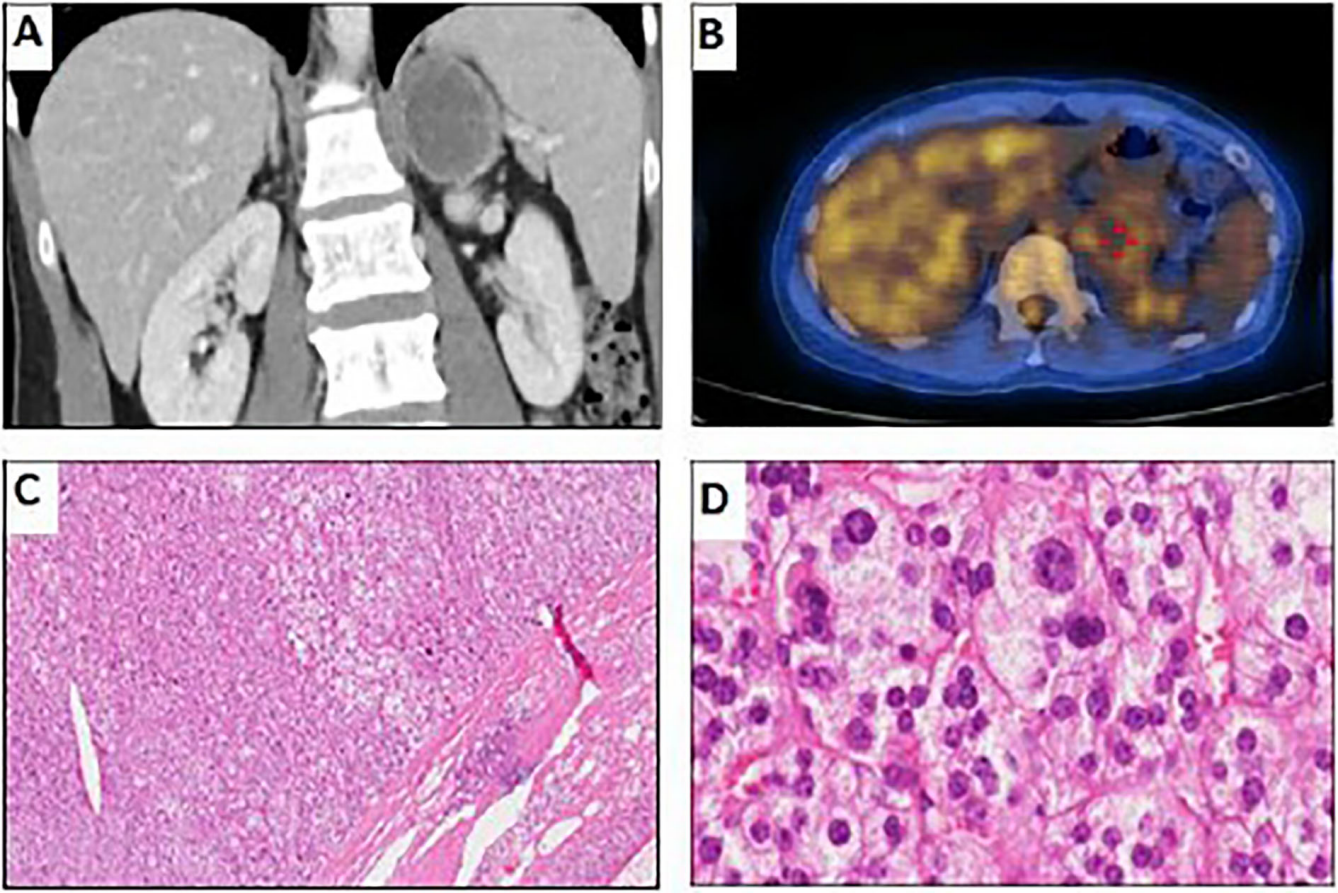
Imaging and pathological examination of the adrenal tumor. **(A)** Coronal CT with contrast showing left adrenal mass approximately 4.5 × 4.8 cm in size with heterogeneous progressive enhancement. **(B)** FDG-PET of the left adrenal gland (early SUVmax, 3.56; delayed SUVmax, 3.25). **(C)** Hematoxylin and eosin stain of adrenal tumor, 40× magnification, well-defined cell borders without capsular invasion. The tumor cells are slightly larger than the surrounding normal adrenal cortical gland cells. **(D)** Hematoxylin and eosin stain of adrenal tumor, 400× magnification, adenoma cells with abundant foamy cytoplasm. Increase of variation in nuclear size with some high-grade nuclear cell (grades 3–4) was seen, but no mitoses, hyperchromisia, or invasion is noted.

**Figure 2:**
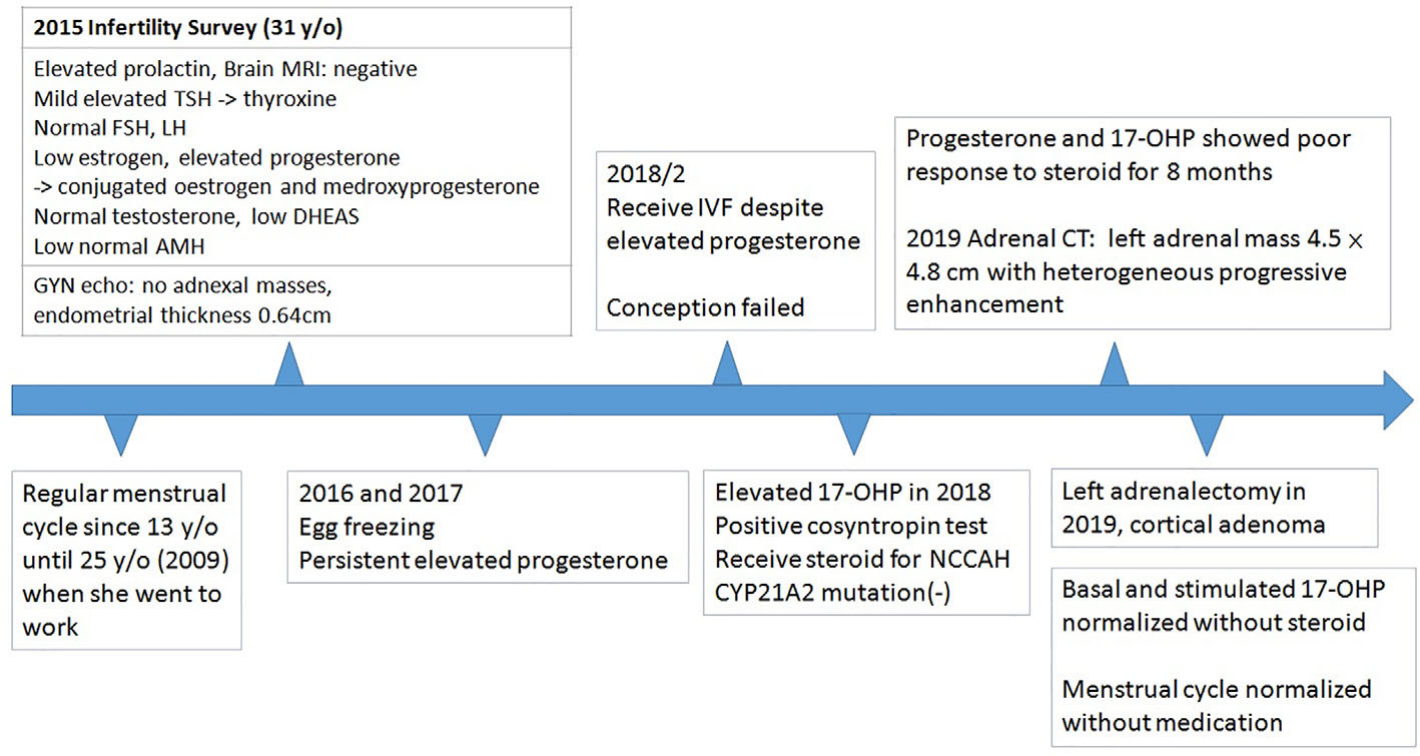
Timeline of the treatment course.

She was referred to our endocrine outpatient clinic in June 2019. Due to elevation of 17-OHP, we arranged computed tomography (CT) which revealed a left adrenal mass approximately 4.5 × 4.8 cm in size with heterogeneous progressive enhancement. Fluorodeoxyglucose positron emission tomography (FDG-PET) revealed a left adrenal gland with an early maximal standardized uptake value (SUV max) of 3.56 and delayed SUV max of 3.25. The adrenal hormone profile were listed in **Table 1**. She underwent laparoscopic left adrenalectomy and pathological examination revealed an adrenal cortical adenoma (**Figure 1**). 17-OHP decreased to 0.5 ng/ml postoperatively. Progesterone declined from 15.3 to 0.1 ng/ml. The cosyntropin stimulation test of 17-OHP was 0.5 and 0.91 ng/ml at 0 and 60 min, respectively (**Table 2**). The irregular menstrual cycle normalized without medication. A 17-OHP autonomous secreting tumor rather than NCCAH was diagnosed due to no more elevation of 17-OHP after adrenalectomy. The timeline of treatment course was showed in **Figure 2**.

**Table 2:** Laboratory result of the cosyntropin stimulation test.

	Pre-adrenalectomy 0 min	Pre-adrenalectomy 60 min	Post-adrenalectomy 0 min	Post-adrenalectomy 60 min
Cortisol (µg/dl)	1.73	9.54	13.23	21.14
17-OHP (ng/ml)	11.3	31.8	0.50	0.91
Progesterone (ng/ml)	2.59	35.4	0.1	0.3

## Discussion

We presented a 36-year-old woman with a history of infertility and initial diagnosis of NCCAH, while CYP21A2 mutation was negative. However, dexamethasone failed to suppress progesterone and 17-OHP levels. CT revealed an adrenal tumor. After adrenalectomy, 17-OHP and progesterone levels declined to within the normal range, with a normal response to cosyntropin stimulation test. In CAH patients with 21-hydroxylase deficiency or 11β-hydroxylase deficiency, DHEA and androstenedione elevation should be detected. On the contrary, 17α-hydroxylase deficiency in CAH patients result in cortisol deficiency, mineralocorticoid excess, and hypogonadism. Elevated DHEA with low androstenedione and 17-OHP may result from 3β-hydroxysteroid dehydrogenase (3β-HSD) type 2 deficiency (5). Despite high progesterone and 17-OHP level, our patient had normal testosterone and low DHEAS level. Hence, CAH related to 21-hydroxylase or 11β-hydroxylase deficiency was not likely. Despite low DHEAS, aldosterone was within normal range in our patient, so 17α-hydroxylase deficiency was not likely, either. Since either testosterone or DHEA, DHEAS, and androstenedione were all not elevated, ovary or adrenal origin androgen secreting tumor were not likely (6). Therefore, an autonomous 17-OHP secreting adrenal tumor was diagnosed.

Most NCCAH patients are diagnosed in adolescence and adult life with hirsutism, acne, and fertility issues (7). Long-term treatment with glucocorticoids will improve the androgen symptoms but may result in long-term complications (7). Oral contraceptive pills are a common treatment option for young females with NCCAH (7). Most women with NCCAH get pregnant smoothly. However, the rate of miscarriages is lower in women who received glucocorticoid treatment (6.5%) than those who did not receive steroid treatment (26.3%) (8). In patients with known classic CAH, 11–58% will have at least one adrenal nodule detected by imaging, and an even higher prevalence was found in an older cohort (82%) (9). Poor response to steroid for CAH/NCCAH patients has been reported. Hua-dong Chen et al. presented three Chinese female patients (9 to 15 years of age) diagnosed with CAH. They all had elevated 17-OHP and androstenedione and suffered from poor response to maximum dose steroid treatment. Ovarian adrenal rest tumors with adrenal hyperplasia were diagnosed later. All patients underwent resection of ovarian tumors. One patient still suffered from unsatisfactory endocrinology markers, which recovered after unilateral adrenalectomy (10).

For all 17-OHP secreting adrenal tumors, gene analysis of CYP21A2 should be performed. For CYP21A2 carriers, it is prudent to offer genetic counseling of the partner, in order to predict the risk of classic CAH in their offspring (8). Reports of 17-OHP secreting adrenal tumors without typical feature and genotype of CAH/NCCAH are increasing (2), while the incidence is still unknown. Since elevation of basal 11-deoxycortisol and 11-deoxycorticosterone have also been noted in AIs (3), impairment of 11 beta-hydroxylase may be one of the reason of these tumors. Since follow-up laboratory tests after adrenalectomy often show no more elevation of progesterone nor 17-OHP even without steroid treatment, the mechanism of adrenal tumor may be different from CAH. On the contrary, some patients who underwent adrenalectomy still had abnormal elevation of 17-OHP (2), and there is still lack of long-term follow-up of these patients who had normal response to ACTH after adrenalectomy, an unknown gene mutation merit further study.

Infertility is worrisome for many women worldwide and the repetitive fertility treatments are exhausting. Physicians should check 17-OHP for infertility workup if persistent high progesterone was detected. Elevated 17-OHP level requires ACTH stimulation test. To ascertain CAH, physician should conduct gene mutation analysis if available. If there is no typical feature and the patients have poor response to steroid treatment, adrenal and ovarian imaging should be arranged. Adrenalectomy may help to suppress 17-OHP and progesterone. Since 17-OHP may be correlated to tumor size, whether 17-OHP can be a marker to predict tumor growth warrant further research.

## Conclusion

When biochemically diagnosed NCCAH is diagnosed, gene mutation analysis should be performed if available. If the patients suffer from poor response to steroid supplementation, the physician should consider adrenal and ovarian imaging. Adrenalectomy may demonstrate promising improvement in suppression of 17-OHP and progesterone.

## Ethics Statement

Written informed consent was obtained from the patient for the publication of any potentially identifiable images or data included in this case report.

## Author Contributions

W-HT: writing and literature search. C-HW, S-HD: analysis and interpretation. C-HT, C-HW: medical and surgical practices. Y-HZ: concept, design, and medical practice.

## Conflict of Interest

The authors declare that the research was conducted in the absence of any commercial or financial relationships that could be construed as a potential conflict of interest.
